# Telemedicine-Supported Hybrid Primary Care in Rural Areas with Healthcare Workforce Shortages: A Qualitative Study from the Heves District, Hungary

**DOI:** 10.3390/healthcare14172727

**Published:** 2026-08-26

**Authors:** Viktor Rekenyi, János Sándor, Ferenc Nagy, Csongor István Szepesi, Nóra Horváth, Yoram T. Kohut, Dániel Eörsi, Anita Pálinkás, Janka Juhász, Hunor Györpál, László Róbert Kolozsvári

**Affiliations:** 1 Doctoral School of Health Sciences, University of Debrecen, 4032 Debrecen, Hungary; 2 Department of Family and Occupational Medicine, Faculty of Medicine, University of Debrecen, 4032 Debrecen, Hungary; 3 Department of Public Health and Epidemiology, Faculty of Medicine, University of Debrecen, 4012 Debrecen, Hungary; 4 Hungarian Charity Service of the Order of Malta, 1011 Budapest, Hungary; 5 Naszlady Attila Health Promotion Program, Hungarian Order of Malta, 1011 Budapest, Hungary; 6 Mental Health Sciences Division, Doctoral College, Semmelweis University, 1085 Budapest, Hungary; 7 Faculty of Medicine, University of Debrecen, 4032 Debrecen, Hungary

**Keywords:** telemedicine, primary care, focus group, qualitative research, nurse triage, physician shortage, Hungary, hybrid care model, health literacy, permanently vacant practices

## Abstract

**Highlights:**

**What are the main findings?**

- A telemedicine-supported hybrid care model combining on-site nurse triage with remote physician consultations was perceived by participating professionals to improve patient safety and care access in permanently vacant Hungarian general practices, potentially reducing reliance on emergency services.
- Successful implementation was perceived to be hindered by patients’ limited familiarity with triage-based care pathways, unsustainable administrative burdens from dual paper-digital documentation, and unresolved IT/software integration challenges.

**What are the implications of the main findings?**

- The future national scalability of hybrid telemedicine care models would likely require concurrent modernization of nursing education curricula to include telemedicine competencies and advanced triage skills.
- Sustainable implementation demands legislative-level resolution of medical software interoperability, reduction in administrative burden, and systematic community health literacy programs.

**Abstract:**

**Background/Objectives:** Primary healthcare in Hungary faces acute physician shortages; in the Heves district, eight general practices are permanently vacant, leaving 11,191 residents without continuous care. In September 2025, the Hungarian Charity Service of the Order of Malta launched the Heves Haziorvosi Pilot (HHP), combining on-site nurse triage with remote telemedicine consultations. This study qualitatively evaluated its implementation and early operation. **Methods:** Three focus group discussions were held in two rounds, before and after implementation, with all 19 professionals delivering the programme (10 practice nurses, 4 general practitioners, 5 degreed professionals). Verbatim Hungarian transcripts were analysed in NVivo 15 (Lumivero, Denver, CO, USA) using the thematic analysis framework of Braun and Clarke with hybrid deductive-inductive coding by three independent researchers until full consensus. **Results:** Daily physician availability rose from approximately 2 to 8 h, and one remote physician concurrently supported four practices. Participants reported reduced professional isolation among nurses, longer and more focused consultations, and perceived, though unquantified, decreases in non-urgent ambulance use. Implementation was constrained by duplicate paper and digital documentation, patients’ limited familiarity with triage, and unresolved software interoperability and IT ergonomic problems. **Conclusions:** The hybrid model was perceived as operationally feasible; national scaling would require nursing curriculum reform, administrative simplification, interoperable medical software, and community health literacy programmes.

## 1. Introduction

Primary healthcare systems worldwide are facing severe challenges due to an aging medical workforce and a growing shortage of physicians [1,2]. One of the most promising tools to mitigate this crisis, widely supported by international research, is telemedicine, which has been shown in a range of settings to bridge geographical distances and to mitigate the shortage of general practitioners (GPs) in rural and disadvantaged areas, while simultaneously enabling the available physician workforce in urban and central regions to extend their reach remotely to underserved communities. The effectiveness of modern telemedicine systems relies heavily on advanced digital diagnostic tools [3,4,5]. The literature indicates that wireless digital stethoscopes used by on-site nursing staff allow for real-time, high-quality auscultation (heart and lung sounds) by a remote physician. Similarly, examinations conducted using camera-assisted (digital) otoscopes, multi-channel ECG devices, 24 h ambulatory blood pressure monitors (ABPMs), and smart blood pressure monitors offer a high level of diagnostic reliability and effectively support medical decision-making in remote care [6,7]. When utilized by Advanced Practice Nurses (APNs) or trained assistants, these devices enable high-quality acute care and the remote management of chronic diseases (e.g., hypertension, cardiovascular conditions), thereby ensuring continuity of care and patient safety even in vacant practices [8].

The Hungarian situation is not exceptional. Rural and socioeconomically disadvantaged areas across high-income health systems face a common constellation of an ageing general practice workforce, insufficient recruitment of younger physicians, and a maldistribution of provision that leaves peripheral regions least well served [1,2]. Comparable responses have been trialled internationally, including task shifting to advanced practice nurses across OECD countries [9], telehealth expansion in remote Australia [10], and structured teleconsultation programmes across Europe [11]. What varies between systems is less the nature of the shortage than the regulatory and financing arrangements that determine which organisational responses are permissible. The Hungarian case is therefore instructive beyond its national context: the constraints identified here, concerning documentation, software interoperability, and nursing scope of practice, are the constraints that any comparable system must resolve.

In Hungary, access to primary care shows significant territorial inequalities, which are further exacerbated by the aging demographic structure of GPs and healthcare professionals, as well as the general shortage of doctors [12]. Nationally, as of early 2025, approximately 956 of Hungary’s 6421 contracted general practices-representing approximately 15% of all primary care districts-were vacant according to National Health Insurance Fund (NEAK) data, with shortages disproportionately concentrated in rural and economically disadvantaged regions [13]. In the Hungarian healthcare system, primary care is provided free of charge by GPs who generally act as gatekeepers to specialized care. Local governments designate primary care districts, and while patients have the right to choose their doctor, GPs are not allowed to refuse patients from their assigned district. To operate in a district with a territorial supply obligation, GPs must purchase a licence, meaning that practices are bought and sold under the control of local governments. Practice financing is dominated by a direct wage support element introduced in 2021 that is determined solely by years of service (40–50% of revenue), with fixed baseline support, a performance-indicator component, and capitation accounting for the remainder [14]. However, one of the most severe problems in the domestic system is the growing number of vacant and permanently vacant general practices-officially defined as districts without a permanent GP for more than six months. Despite government financial subsidies, these unfilled practices remain mostly concentrated in remote, less-developed areas of the country. When a practice becomes vacant, it is typically covered by deputizing GPs from neighbouring districts, creating a significant burden on both sides: deputy doctors must travel extensively to provide intermittent care, while patients are left without daily local access to a physician [15,16].

Alternatively, since local governments are legally obligated to ensure continuous healthcare provision for their residents, they can enter into a direct contract with a GP to provide long-term substitution. Under this arrangement, the physician is not required to purchase the practice licence; nevertheless, the district officially remains classified as ‘permanently vacant’ in the national registry [17]. This substitution model is often unstable: professionals frequently do not stay long-term, and even when they do, the limited consultation time-in vacant practices consistently restricted to just 2 h per day on average (NNGYK [Nemzeti Népegészségügyi és Gyógyszerészeti Központ; National Centre for Public Health and Pharmacy] operating licences for vacant practices stipulate a minimum of only 10 consultation hours per week)-is strictly sufficient only for the immediate, firefighting-style treatment of acute patients, alongside unavoidable administrative duties such as issuing sick-leave certificates and prescriptions [15,18]. Consequently, prevention and the regular management of chronic patients are marginalized; the doctor simply lacks the time, and due to constant turnover or time constraints, they cannot get to know their patients well enough to provide continuous, proper general practice care in the traditional sense.

The continuously overburdened outpatient and hospital specialist care, combined with these shortcomings in primary care, justify a structural transformation of the current general practice system. Due to the unfavourable characteristics of the GP age pyramid-marked by a disproportionately large cohort of physicians approaching retirement age and an insufficient intake of young doctors-the anticipated worsening shortage of GPs cannot be solved simply by replacing retiring doctors; sustainable operation requires new types of care formats and innovative care organization solutions [19,20].

This problem particularly affects the Heves district, located in north-eastern Hungary. Based on April 2025 data from the National Health Insurance Fund (NEAK), the district, which serves a population of 34,468, has a total of 23 practices, 8 of which (one paediatric and seven mixed-practice districts (i.e., practices serving both adult and paediatric patients) are permanently vacant. The population of the vacant mixed districts totals 11,191 people, posing a critical challenge to the region’s healthcare system [13,21].

Table 1 situates the study setting. The Heves district ranks as the ninth least developed district among the 197 districts of Hungary and is one of only 36 designated for the complex development programme of the government, the most intensive category of statutory territorial support [22]. Against this background of structural disadvantage, the proportion of practices without a permanent general practitioner is 2.3 times the national figure, and 11,191 of the 34,468 residents of the district live in such a practice area [13,21]. The Heves district therefore concentrates precisely those socioeconomic and workforce conditions that make general practice least attractive to physicians. It represents not an atypical outlier but an advanced case of a nationwide trajectory, an informative setting in which to test organisational responses before comparable conditions become widespread elsewhere.

In response to this crisis, the Hungarian Charity Service of the Order of Malta launched the Heves Haziorvosi Pilot (HHP), an integrated district-level programme combining in-person nurse triage with remote telemedicine consultations. The HHP was formally launched on 1 September 2025. Prior to this formal launch, a preparatory pilot phase had already been implemented in the preceding months; experiences and data from this phase informed the design of the present study. The goal of the HHP is to develop a hybrid primary care model that builds upon existing, often fragmented substitution structures to safely organize care for patients in permanently vacant practices. In this model, substituting GPs provide in-person consultations under unchanged contractual frameworks, while care is provided via telemedicine during time slots when a doctor is not physically present. Telemedicine workstations, digital stethoscopes, 12-lead ECGs, and ankle-brachial index meters were deployed to the clinics. The key actor in this new type of care organization is the local practice nurse, who performs nurse triage and examination preparation based on dedicated protocols. If a case cannot be resolved within the nurse’s scope of practice, the telemedicine physician makes a decision during a remote consultation. As a safety net for the model, a Central Clinic (HKR) was established in the town of Heves, operating on weekdays (between 8 a.m. and 4 p.m.), where in-person contact examinations are provided if professionally justified.

### Research Gap and Study Rationale

Three strands of evidence bear on the model examined here, and each stops short of it. First, nurse-led teletriage is well established as a safe means of expanding access; a recent synthesis concluded that properly implemented systems do not increase mortality, hospitalisation, or emergency department referral rates [23]. That evidence base, however, describes predominantly telephone-based triage conducted within practices that retain their own physician, with the nurse assessing remotely and directing the patient onward rather than acting as the on-site diagnostic proxy of the physician. Second, European task-shifting models that combine delegation with telecare devices, most notably the German AGnES programme, in which general practitioners delegated home visits to qualified practice nurses equipped with remote monitoring devices, were designed to relieve practising physicians of workload rather than to compensate for a physician who is structurally absent [24]. Third, evidence on primary care digitalisation from Central and Eastern Europe remains scarce and largely descriptive; the multi-country PRICOV-19 study identified governmental and infrastructural support, rather than the clinical organisation of care, as the principal determinant of adoption across the region [25].

No published account therefore describes the configuration that the HHP represents: a permanently vacant practice in which an on-site nurse performs protocol-based triage and device-assisted physical examination while a geographically remote physician retains clinical responsibility, operating within a publicly financed European health system. The present study addresses this gap in three ways: it documents how such a model functions in daily practice from the perspective of the professionals who deliver it; it identifies the organisational, educational, and regulatory conditions that govern its sustainability; and it thereby provides a transferable account for health systems facing comparable general practitioner shortages.

This study aimed to explore the experiences and perceptions of healthcare professionals regarding the implementation of a nurse-led hybrid telemedicine model in primary care practices with permanent vacancies. Within this aim, six dimensions were examined during the implementation period between June 2025 and January 2026: access to care, including appointment scheduling, waiting times, and travel burden; the quality and organisation of care, including the transparency of patient pathways; the practical effects of introducing telemedicine and nurse triage; the coordinating role of the Central Clinic; professional support and teamwork among healthcare workers; and the doctor-patient relationship, communication, and patient health literacy.

## 2. Materials and Methods

### 2.1. Study Design and Reflexivity

The study employed a qualitative descriptive design in the tradition described by Sandelowski [26], an approach that stays close to the accounts given by participants and seeks a comprehensive summary of events in the everyday terms used to describe them, rather than the theoretical abstraction sought in grounded theory or the essence sought in phenomenology. This orientation was chosen because the object of inquiry is a concrete organisational intervention: the aim was to establish how the model operates in practice, what facilitates and impedes it, and how it is experienced by those delivering it, all of which call for a descriptive rather than a theory-generating account. This study is reported in accordance with the COREQ (Consolidated Criteria for Reporting Qualitative Research) 32-item checklist for qualitative interview and focus group studies [27]. Although the HHP preparations began prior to the formal start of data collection, all research interviews and focus group sessions were conducted following receipt of formal ethical approval (ETT TUKEB, protocol code BM/25735-3/2025, approved 22 October 2025). To reduce the risk of social desirability bias, all interviews and focus groups were conducted by university researchers with no contractual or hierarchical relationship with the operating organization; participants were assured of confidentiality, and their employer was not informed of their individual decisions regarding participation. Patients were not included in the present analysis; the exploration of patients’ perspectives, foreseen in the approved research protocol, is planned as a separate study. Audio recordings of all focus group discussions were transcribed verbatim. Since the original interviews were conducted in Hungarian, the primary data analysis and coding were performed in the source language to preserve contextual and cultural nuances. The textual data were analyzed using qualitative thematic analysis, adhering to the foundational methodological framework established by Braun and Clarke [28]. NVivo (version 15; Lumivero, Denver, CO, USA) qualitative data analysis software was utilized for data management, coding, and running queries [29]. A hybrid coding strategy was employed during the analysis, incorporating both deductive and inductive approaches [30].

Regarding reflexivity, none of the research team members hold a legal or contractual relationship with the Hungarian Charity Service of the Order of Malta operating the HHP. The principal investigator is affiliated with the University of Debrecen, Department of Family Medicine and Occupational Health, and has no hierarchical authority over the participating nurses or GPs. To minimise potential interpretive bias, all researchers disclosed their prior familiarity with the Heves district context at the outset of the study, and the coding and thematic interpretation were conducted independently by three researchers (V.R., Cs.I.Sz., N.H.) before consensus discussions.

All three researchers who performed the coding (V.R., Cs.I.Sz., N.H.) are physicians trained in family medicine. This shared professional background carried two identifiable risks for interpretation. First, a coding team composed exclusively of physicians may be predisposed to read nursing accounts through a medical frame of reference and to underweight concerns specific to nursing practice. Second, none of the team held a sceptical prior position towards telemedicine; all regarded technology-supported care as a plausible response to workforce shortage, creating a risk that ambiguous statements would be read favourably. Three measures were adopted to limit these risks. Coding was performed independently before any joint discussion, so that initial interpretations were not shaped by consensus pressure. Codes were required to be grounded in verbatim material, and disagreements were resolved by returning to the transcript rather than by seniority. Finally, the inductive strand of the codebook was deliberately left open, so that concerns raised by participants which did not correspond to the expectations of the research team could be entered into the analysis on their own terms. The two findings that most constrain a favourable reading of the model, namely the scale of the administrative burden and the extent of patient resistance, both emerged inductively and were not anticipated.

Written informed consent was obtained from all participants before the start of each focus group discussion. During the discussions, the moderator periodically summarised the points that had been raised and invited participants to confirm or correct that summary, providing respondent validation within the sessions. No post-hoc member checking was undertaken: transcripts were not returned to participants for correction, and the final thematic structure was not submitted to them for comment. This is acknowledged among the limitations in Section 4.5.

### 2.2. Sampling Strategy and Data Collection

A total population sampling strategy was applied: rather than drawing a sample, every healthcare professional delivering care within the HHP was eligible and was invited to participate. Recruitment took place through personal briefings at the participating practices and by telephone. All 19 eligible professionals, comprising 10 practice nurses, 4 general practitioners, and 5 degreed professionals (an Advanced Practice Nurse, a social worker, and a practice manager), agreed to take part, yielding a participation rate of 100% with no refusals and no dropouts. The participating group therefore constitutes the complete population of professionals operating the programme rather than a subset of it, so selection bias arising from differential willingness to participate does not apply.

Data were collected in two rounds through three focus group discussions, each held at a general practice surgery in the town of Heves and lasting approximately 90 min. The first round preceded the formal launch of the HHP and captured baseline conditions and expectations. To allow candid discussion within peer groups and to avoid the constraining effect of professional hierarchy, this round was conducted as two parallel groups: general practitioners together with degreed professionals; practice nurses. The second round was held after the programme had been in operation and brought general practitioners and nursing staff together to reflect on implementation experience. All participants took part in both rounds. No individual interviews were conducted and no sessions were repeated. This pre- and post-implementation structure permits expectations to be compared directly against subsequent experience within the same professional population, and this is reflected in the organisation of the Results.

The adequacy of this design was appraised against two criteria. First, the information power framework of Malterud et al. [31] holds that fewer participants are required where the study aim is narrow, the sample highly specific, the dialogue of high quality, and the analysis cross-case. All four conditions were met: the aim was confined to a single standardised intervention; every participant had direct operational experience of it; the discussions were led by experienced qualitative researchers using a piloted semi-structured guide; and the analysis was thematic and cross-case rather than case-by-case. Second, empirical work on focus group sample sizes indicates that more than 80% of themes are discoverable within two to three groups, and that three groups are sufficient to identify all of the most prevalent themes in a data set [32]. Consistent with this, coding of the final transcript generated no new codes, indicating that the material was sufficient for the analytical aim.

### 2.3. Study Setting and Deployed Technology

Although the original objective was to extend the programme to all eight permanently vacant practices, the HHP was implemented in four satellite GP practices (Tiszanána, Kömlő, Kisköre, and Átány), complemented by the Central Clinic (HKR) in the town of Heves. Each practice was equipped with a standardised telemedicine workstation providing device-assisted physical assessment: a Thinklabs One digital stethoscope (Thinklabs Medical LLC, Greenwood Village, CO, USA) for remote auscultation of heart and lung sounds, a Firefly DE500 digital video otoscope (Firefly Global, Belmont, CA, USA) for examination of the ear, MESI mTABLET integrated diagnostic modules (MESI d.o.o., Ljubljana, Slovenia) for 12-lead electrocardiography, ankle-brachial index measurement and blood pressure measurement, and a Kameleon teleconsultation camera (Kameleon Kamera Kft., Budapest, Hungary). Full technical specifications are listed in Table S1 of the Supplementary Materials [33,34,35,36]. Remote physician consultations were conducted via a dedicated telemedicine platform using these devices, operated by the on-site nursing staff. Prior to the launch, participating nurses received structured preparatory training covering the use of the telemedicine devices, the triage protocol, and the new documentation requirements. Each patient contact began with a standardized triage sheet guiding protocol-based questioning and mandatory basic measurements (e.g., blood pressure). Care episodes were documented in parallel in the electronic medical record system and on paper-based forms. After each video consultation, a structured feedback routine was applied: the nurse and the patient jointly summarized the information discussed to verify accurate understanding.

Responsibilities within the model were allocated as follows: The practice nurse is the first point of contact: she conducts protocol-based triage, performs the mandatory baseline measurements and any device-assisted examination requested, prepares the case for the remote consultation, and afterwards verifies the understanding of the patient of the instructions given. Within her scope of practice she resolves cases that do not require medical decision-making, principally repeat prescriptions and routine documentation. The telemedicine physician, working from the Central Clinic, assumes clinical responsibility for every case escalated to him: he conducts the remote consultation, interprets the transmitted findings, and decides on treatment, referral, or the need for in-person examination. The substituting general practitioner continues to provide in-person consultations at the satellite practices under unchanged contractual arrangements. The Advanced Practice Nurse supports chronic disease management and supervises the professional development of nursing staff across the participating practices, while the social worker addresses the non-clinical barriers to care that arise in a disadvantaged population, and the practice manager coordinates scheduling and the interface with the operating organisation. Dedicated practice administrators, introduced during the implementation period, took over non-clinical documentation so that nursing staff could concentrate on clinical and triage tasks.

The model incorporates a defined escalation pathway. Where the triage assessment of the nurse or the subsequent remote consultation indicates that direct physician examination is required, the patient is referred to the Central Clinic, where in-person examination is available on weekdays between 8 a.m. and 4 p.m. Transport to the Central Clinic is provided by the patient transport service operated by the Order of Malta, so that the availability of in-person medical assessment is not conditional on the means of travel of the patient.

### 2.4. Data Analysis

To ensure analytical rigor and minimize individual researcher bias, the coding process and the development of the codebook were collaboratively conducted by three independent researchers (V.R., Cs.I.Sz., N.H.). The backbone of the codebook consisted of pre-defined deductive codes based on the semi-structured interview guide (e.g., access to care, telemedicine application, patient pathway organization, professional support, triage system). During the iterative, open reading and coding of the transcripts, this framework was supplemented with inductive codes that emerged spontaneously from the data. These codes captured prominent daily experiences introduced by the interviewees themselves (e.g., IT and technical barriers, increasing administrative burden, health literacy and fear). The analysis proceeded in five stages: First, each researcher read the full set of transcripts without coding in order to become familiar with the material as a whole. Second, each researcher independently coded the transcripts line by line, applying the pre-defined deductive codes where the material corresponded to them and generating new inductive codes where it did not; inductive codes were formulated in terms close to the language used by participants. Third, the three independently produced coding frames were compared in consensus meetings, in which discrepancies were resolved by returning to the transcript, overlapping codes were merged, and code definitions were sharpened until the same segment would be coded identically by all three researchers. Fourth, the agreed codes were grouped into candidate themes on the basis of shared meaning rather than shared vocabulary, and each candidate theme was checked against the coded extracts and against the full data set to confirm that it was adequately supported and internally coherent; two initial candidate themes were discarded at this stage because they proved to be facets of others. Fifth, the final themes were named and defined, and the boundaries between them were agreed by the three researchers. Coding of the final transcript generated no new codes.

To ensure transparency and replicability, the finalized deductive codebook is detailed in Table 2, while the inductive codes that emerged from the data are presented in Table 3. To ensure that the perspectives of different care levels and stakeholders could be differentiated and compared, the extracted quotes were categorized by roles (Cases/Case Classifications: doctors, nurses, assistants, degreed professionals) within the software. This classification enabled coded segments to be retrieved and compared by professional group, so that points of consensus and of tension between general practitioners, nursing staff, and degreed professionals could be examined systematically rather than impressionistically. These comparisons were interpretive: coding frequencies were not treated as quantitative indicators. For the purpose of academic reporting, the finalized codebooks and selected representative quotes were subsequently translated into English, with a rigorous cross-checking process to ensure semantic equivalence and validity.

### 2.5. Analytical Rigour and Trustworthiness

A qualitative design was selected because the research question concerns how a novel model of care organisation is enacted and experienced in daily practice, a question of mechanism and meaning rather than of magnitude. Quantitative evaluation of the effects of the programme on service utilisation, clinical outcomes, and cost-effectiveness is planned as a separate study once a sufficient observation period has accrued; the present findings are intended to generate the hypotheses that such an evaluation will test. Statistical testing of coding frequencies was deliberately not undertaken, as contributions within a focus group are not independent observations and the frequency with which a topic is raised reflects group dynamics as much as its substantive importance.

Rigour was accordingly assessed against the criteria established for qualitative inquiry [37]. Credibility was addressed through independent coding by three researchers, iterative consensus meetings continued until no unresolved discrepancies remained, and the presentation of verbatim quotations allowed readers to judge the fit between data and interpretation. Dependability was supported by a pre-specified deductive codebook derived from the interview guide (Table 2), together with full documentation of the inductive codes that emerged during analysis (Table 3), rendering the analytical pathway traceable. Confirmability rests on the reflexivity procedures described in Section 2.1: no member of the research team held a contractual relationship with the operating organisation, and prior familiarity with the district was disclosed at the outset. Transferability is supported by the detailed account of the setting, the intervention, and the deployed technology given in Section 2.3, which enables readers to assess applicability to their own contexts. Reporting follows the 32-item COREQ checklist [27].

## 3. Results

The analysis produced four themes, each comprising two subthemes, derived from the deductive and inductive codes listed in Table 2 and Table 3. The final thematic structure and the codes contributing to each theme are set out in Table 4. The findings are reported in two parts, corresponding to the two rounds of data collection: Section 3.1 presents the baseline situation and the expectations expressed before implementation, and Section 3.2 presents the experiences reported after the programme had been in operation.

Representative participant quotations illustrating each deductive and inductive code are presented in Table 5.

### 3.1. The Baseline State of Primary Care and Expectations for the Hybrid Model

#### 3.1.1. Access to Care and Current Patient Pathways

The current operation of vacant practices is experienced by professionals as a form of constant crisis management. Due to the structural limitations of the substitution system, access to care has significantly narrowed. Respondents unanimously stated that “in long-term substituted practices, there are practically two to three hours of active consultation time”, which one interviewed doctor simply referred to as “firefighting”. The limited physical presence of doctors has also distorted patient pathways: patients often “gather at 8 o’clock, standing there beautifully in the doorway, like little birds, sparrows, waiting for the door to open”. Outside the short consultation hours, patients experience a sense of being unprovided for, forcing them to turn to on-call services or unnecessarily to the ambulance service with acute problems.

Professionals expect a significant improvement in access and patient pathways from the HHP, especially from the Central Clinic’s (HKR) opening hours from 8 AM to 4 PM. Expectations suggest that patients will be retained within the primary care system. Hopes tied to the new system indicate that, through local telemedicine points, patients in the future “will not have to go to another city,” which will be a massive relief, especially for elderly patients who have difficulty traveling. As one professional vividly summarized: “They do not have to travel to another town. From the more remote settlements, people would not go all the way to Eger. If I have some problem, I go in to the nurse I know, have a telemedicine consultation, and I am talking to a doctor.”

#### 3.1.2. Professional Support and the Human Resources Crisis

One of the most severe problems of the current system is the overload and professional isolation of assistants and nurses. Nurses in substituted districts “were always alone” in making daily decisions. The interviews highlighted the “inhumane” burden placed on healthcare workers during daily operations: “during consultation hours, the patient is constantly sitting here, and the phone is ringing… doing work in parallel”. The primary positive expectation regarding the HHP is networking and the establishment of a continuous medical background. Nurses expect relief from the fact that, through the telemedicine connection, “during standby time, you don’t have to hunt for the doctor… immediately, even if only through a camera, but there is a doctor”.

#### 3.1.3. Telemedicine and Triage: Benefits and Preliminary Concerns

While the introduction of telemedicine and structured triage is evaluated by professional management as an increase in patient safety, interviewees forecasted serious administrative and technical difficulties regarding practical implementation. Nurses projected that a previously routine, 1 min phone prescription would “stretch to 5–10 min” due to the new, mandatory data collection form (triage sheet). Professionals also noted the lack of a central medical software and the continuous “logging in and out” of different systems as a serious barrier. Despite these concerns, doctors remained confident that telemedicine would restructure the current, often unsustainable “telephone healing” and bring about “a significant improvement in patient safety”. One professional highlighted the information gap that this creates: “They [the on-call doctor] cannot see into the system at all-they have no idea what the patient’s underlying conditions are. The patient either knows, or does not, or only partially knows. There is simply no way to retrieve the relevant parameters.”

#### 3.1.4. Doctor-Patient Relationship, Communication, and Health Literacy

One of the greatest social obstacles to the success of the model is the health literacy and fear of the disadvantaged population. The interviews revealed that a segment of the target population tends to postpone seeking medical care, partly owing to financial constraints and partly owing to a lack of information and fear of a potential diagnosis (“terrified to go to the doctor and find out there is a problem”). A further challenge is the traditional, authoritarian doctor-patient relationship, where “the person of the doctor is sacred”, so patients often only follow nursing advice if it is later confirmed by a doctor. Professionals anticipate that the expanded 8 h consultation time will finally allow for genuine patient education, and positive experiences will spread by word of mouth to gradually transform the population’s attitude towards healthcare.

### 3.2. Experiences, Successes, and Challenges in Implementing the Hybrid Model

The analysis of focus group interviews conducted in the period following the launch of the HHP revealed that the introduction of telemedicine and triage-based care brought perceived improvements in quality and patient safety, as reported by participants. However, the new system encountered serious cultural, administrative, and technological obstacles in daily practice.

#### 3.2.1. Predictable Access and Strengthened Professional Support

One of the most tangible successes of the HHP was the substantial expansion of availability. Instead of the previous few hours of substitution per day, the clinics now provide access to telemedicine medical support for eight hours a day. Participants highlighted as a structural advantage that a single telemedicine workstation at the Central Clinic can serve multiple practices simultaneously, enabling one remote physician to support several clinics concurrently. They considered that this represented a substantial saving in physician time compared with deploying a separate physician at each site, although no formal workforce or cost analysis was undertaken in the present study. Some participants reported that daytime on-call service use appeared to have decreased in the affected settlements, citing informal verbal feedback they had received from the National Ambulance Service. This account was not corroborated by service utilisation data, was not quantified in any form, and is reported here solely as a perception expressed by participants rather than as an observed effect of the intervention.

For nurses, continuous professional support substantially reduced their previous professional isolation. They manage critical cases (such as extremely high blood pressure or stomach cramps) more confidently, knowing that a doctor is immediately available via screen. Thanks to structured nursing preparation, doctors have the opportunity to dedicate an uninterrupted “full 100% focused 20 min” to a patient, replacing the previous rushed pace of treating up to 56 patients a day. Participants reported that patients appeared more satisfied with the increased attention and detailed explanations. Participants considered that the quality of care was further supported by the newly introduced feedback routine, in which the nurse and the patient summarise what was discussed after each video consultation in order to verify understanding.

However, the increased responsibility and the use of telemedicine tools also highlighted the shortcomings in nursing education. As one older nurse participant candidly reflected, “We have to learn [to use it]-we can barely switch it on ourselves. For the younger generation it comes naturally, as they were born into it, but we are 60+.” Since the core curriculum of traditional nursing training does not prepare healthcare professionals for telemedicine care and the more independent decision-making expected at this level, both nurses and doctors expressed a strong need for targeted further training, evidence-based curricula, and regular practical exams.

#### 3.2.2. The Price of Structured Care: Administrative Burdens and Triage

Participants described the triage protocol as delivering a professional “health gain”, giving as an example the fact that the omission of a blood pressure measurement no longer occurred; at the same time, they reported that the administrative burden increased substantially. Healthcare professionals consistently described the duality of paper-based and digital recording as “sweat-inducing” and unsustainable in its current form in the long run. The programme management ultimately engaged dedicated practice administrators to handle non-clinical administrative tasks-such as documentation, scheduling, and form processing-thereby allowing nursing staff to focus on their core clinical and triage responsibilities. From the patients’ perspective, triage often causes frustration; many only perceive that they “have to wait longer” and ask “Do we still have to write?”.

#### 3.2.3. Social Resistance and Lack of Health Literacy

The population found it difficult to adapt to the elimination of the decades-old habit of skipping lines. When regulated patient intake was introduced, participants reported that some patients initially responded with frustration to the new appointment and triage procedures, including verbal conflict in some cases. Adapting to the structured system took considerable time. The true goal of the HHP-boosting chronic care management-faced serious obstacles related to patients’ limited familiarity with preventive and triage-based care. Experiences showed that patients were used to firefighting-style care: “as long as it doesn’t hurt, doesn’t sting, they have no complaints, they don’t come”. According to professionals, public information was the weakest point of the HHP; dedicated professionals are needed to systematically explain the operation of the system to the population in local community spaces (elderly clubs, pubs, churches). As one participant described the situation, “Even running a 40-degree fever, if there are 25 people ahead of them, they simply cannot accept that they are 25th in the queue and have to wait for the 24 in front-who also have 40-degree fevers. None of that matters to them, so they just leave.”

#### 3.2.4. Technological, IT, and Systemic Challenges

Although the infrastructural background has been established, daily operations are still slowed down by technical “teething problems.” The ergonomics of the devices are inadequate: nurses constantly have to plug cables “back and forth,” and bandwidth issues are common, preventing the digital stethoscope’s audio and the video feed from being enjoyed simultaneously in good quality. Previously, handling nursing delegation caused an IT anomaly, as the administration ran “under the small name tag of the substitute doctors.” This issue has since been resolved by providing nurses with a separate login interface. A further problem is the lack of active substance-based prescribing, and inter-professional telemedicine communication (e.g., psychiatric, dietetic) remains hindered, requiring completely isolated rooms in the clinics in the future. Notably, despite the detailed exploration of implementation barriers, no participant reported any incident or concern regarding the safety of patient care during the study period.

#### 3.2.5. Differences Between Professional Groups

Although all principal themes were raised by every professional group, the groups framed them at different levels of analysis. Practice nurses discussed the model predominantly in operational terms: the administrative burden was described as personal time loss within the working day, technological difficulty as a matter of handling the devices, and patient adaptation as an interpersonal task accomplished in the consulting room and spread by word of mouth. General practitioners and degreed professionals framed the same phenomena systemically: administrative burden was attributed to the absence of a unified medical software platform, technological difficulty to interoperability between systems, and limited patient adaptation to the socioeconomic determinants of health literacy in a disadvantaged population. Neither framing contradicted the other; each group described the level of the system at which it encountered the problem. This divergence carries a practical implication, in that an intervention directed at one level, such as the engagement of practice administrators, relieves the group experiencing the problem operationally without resolving the structural cause identified by the other.

## 4. Discussion

### 4.1. Main Findings

The early experiences of the hybrid primary care model supported by telemedicine and nurse triage, implemented in the Heves district, suggest that participating professionals perceived the model as a feasible way to support care continuity and patient safety in permanently vacant practices struggling with physician shortages. At the same time, our results make it clear that the mere deployment of technological infrastructure is not enough; successful implementation requires a profound cultural, administrative, and educational paradigm shift at all levels of the healthcare system.

### 4.2. Comparison with the Literature

One of the most important findings of our study is that participants perceived the combined application of structured nurse triage and a telemedicine medical background to have substantially reduced the professional isolation of practice nurses, and considered that it had the potential to support patient safety. This observation strongly aligns with the international literature, which has long emphasized the importance of task shifting and the role of nurses and APNs in rural primary care [9,10,38]. Contemporary research examining the effectiveness of telemedicine also confirms that the use of digital diagnostic tools-such as digital stethoscopes or multi-channel ECGs-with appropriate assistance enables a high level of diagnostic accuracy and reliable remote decision support [3,6,7,10]. Our results complement this from a practical standpoint: participants reported that continuous remote medical supervision had made nurses more confident in managing critical situations, and that it afforded physicians focused, uninterrupted consultation time in place of the previous “firefighting-style” visits.

A recurring concern about remote care is that physical examination cannot be fully replicated at a distance, and that a physician observing a patient in person perceives more than any transmitted signal conveys. This concern is well founded: a systematic review of physical examination components adapted for telemedicine found that although virtual assessments approached in-person accuracy for many individual components, interrater reliability varied widely, and the inability to perform hands-on manoeuvres remains a substantive limitation [39]. The HHP was designed around this constraint rather than in disregard of it. In the hybrid model examined here, the physical examination is not omitted but relocated: the patient attends the practice in person, and a trained nurse conducts protocol-based assessment supported by digital auscultation, video otoscopy, 12-lead electrocardiography, and ankle-brachial index measurement. What is delivered remotely is the clinical judgement of the physician, not the examination itself. Where that judgement indicates that direct examination is required, the patient is transported to the Central Clinic and examined in person. The relevant comparison is therefore not between telemedicine and conventional in-person general practice, but between this hybrid arrangement and the counterfactual that it replaces, a permanently vacant practice with approximately two hours of physician presence per day, in which patients outside that window rely on telephone advice or the ambulance service. Against that comparator, rather than against an idealised fully staffed practice, participants judged the model to improve both access and the quality of clinical assessment.

The most pronounced obstacle identified in the research is the patients’ limited familiarity with preventive and triage-based care, and cultural resistance to structured care. For decades, patients have been accustomed to immediate, out-of-turn care-often solely for medication prescriptions-making the concepts of preventive care and chronic disease management largely unknown to them. Health sociological research frequently points out that the introduction of new health technologies and stricter protocols in disadvantaged regions often initially provokes aggression and frustration if not paired with an appropriate level of education [40,41]. Our findings were consistent with this: there is a need for dedicated professionals (e.g., social workers) who support the population in learning to navigate and use the new care system, explaining its benefits in local community spaces, and shifting the focus from acute care to the management of chronic diseases.

This pattern is more fully explained by Normalization Process Theory, which holds that a new practice becomes embedded in routine work through four mechanisms: coherence, i.e., the sense participants make of it; cognitive participation, i.e., their willingness to engage with it; collective action, i.e., the work required to operationalise it; and reflexive monitoring, i.e., the appraisal of its effects [42]. Read through this framework, the difficulties that we identified were concentrated in the first and third mechanisms, and on opposite sides of the encounter. Among professionals, coherence was achieved readily: staff understood the rationale for triage and engaged with it, and the obstacles that they reported were those of collective action, namely duplicate documentation, fragmented software, and inadequate preparatory training. Among patients, by contrast, coherence itself was absent: structured triage was not understood as a mechanism for allocating clinical attention by need but was experienced as an additional obstacle to a physician, which is why it was met with frustration rather than with the passive non-adherence more usual in this population. This distinction matters for implementation, because the two deficits require different remedies. Deficits in collective action are addressed by resources and system design, whereas deficits in coherence are addressed only by explanation and by the accumulation of direct experience, which is consistent with the emphasis participants placed on community-level information provision and on trust spreading by word of mouth.

While participants regarded the model as a substantial improvement for patients, they also reported that it imposed a considerable administrative burden on the caregiving staff. The mandatory use of triage sheets is professionally justified, but the duality of paper-based and digital recording is unsustainable in the long run. Numerous international health informatics studies warn that a sudden increase in administrative burdens during digital transitions can lead to staff burnout if support personnel and adequate software integration are not provided [43,44]. The HHP management responded correctly by incorporating practice administrators, indicating that a modern primary care team must now also demand dedicated administrative staff.

### 4.3. Implications for Practice and Policy

However, the model also highlighted the structural shortcomings of domestic nursing education. Traditional core curricula currently do not prepare healthcare professionals for the routine use of telemedicine tools and the more independent decision-making expected at this level. For this model to be nationally scalable, it is essential to integrate targeted, evidence-based further training and modules that develop critical thinking into formal educational systems, as advocated by international guidelines on digital health workforce development [45,46].

Technological ergonomics, the fluctuation of internet bandwidth, and the rigidity of medical software-including the difficulties of IT tracking of nursing delegation and the lack of active substance-based prescribing-are macro-level anomalies that must be addressed at the legislative and software development levels in the future [47,48].

The transferability of these findings requires differentiation, as the components of the model are not equally portable. Three elements appear broadly transferable to any system facing a similar shortage: the separation of the physical examination from the clinical decision, which permits a physician to serve several sites without the examination being lost; the pairing of remote consultation with a defined escalation route to in-person assessment, including the transport that makes that route usable; and the recognition that the introduction of triage into a population unaccustomed to it is a communication task requiring dedicated resource rather than a procedural adjustment. Two further elements are strongly context-dependent. The scope of practice permitted to nurses, and the documentation and prescribing requirements imposed on them, are set nationally, and the burden that we identified is a feature of Hungarian regulation rather than of hybrid care as such; systems with broader nursing scope would encounter this constraint in a weaker form, and those with narrower scope could not implement the model at all in its present configuration. Similarly, the model presupposes that a substituting physician and a central clinic already exist within reach, which is a characteristic of the Hungarian substitution arrangement and not a universal feature. The practical implication is that the organisational logic of the model transfers more readily than its specific configuration.

### 4.4. Comparison with Related Implementation Models

To situate these findings and to allow their robustness to be judged, Table 6 compares the HHP with four established models of technology-supported or task-shifted primary care.

Two features distinguish the HHP from these comparators. First, all four established models operate in settings where the practice retains its own general practitioner; the HHP operates in practices that have none, so the model does not supplement medical presence but constitutes it. In-person physician assessment is not eliminated, however, but escalated: where the remote physician judges direct examination necessary, the patient is transported to the Central Clinic for face-to-face assessment, so the model retains a defined pathway to in-person medical care rather than dispensing with it. Second, in models that rely on remote assessment, the physical examination is either omitted or transferred to the patient; in the HHP, it is retained at the point of care and performed by a trained nurse with diagnostic device support. Together these features define the contribution of the present study.

Our findings converge with the international literature on several points, which supports their external validity. The reduction in professional isolation among nurses and their increased confidence in managing acute presentations correspond to outcomes reported in systematic reviews of nurse substitution in primary care [9,38]. The administrative burden generated by parallel paper-based and digital documentation reproduces a pattern documented repeatedly in the health informatics literature on digital transitions [43,44], and the software interoperability and ergonomic problems identified here match the barriers catalogued in a worldwide review of telemedicine adoption [47].

One finding diverges from expectation. Evaluations of large telehealth programmes have frequently identified professional scepticism and staff resistance as principal obstacles to adoption [40]. In the HHP, resistance came predominantly from patients rather than from professionals, and staff assessments of the model were consistently favourable. The most plausible explanation lies in the counterfactual against which participants judged the intervention: in a practice that had operated for years with approximately two hours of daily physician presence, any arrangement securing continuous medical availability is likely to be welcomed rather than resisted. This suggests that professional receptiveness to telemedicine is conditioned less by the technology itself than by the adequacy of the arrangement it displaces, a consideration relevant to implementation planning in comparably underserved settings elsewhere.

### 4.5. Strengths and Limitations

A key strength of this study is its hybrid deductive-inductive analytical approach, which enabled both the systematic assessment of pre-defined implementation domains and the identification of unanticipated factors. To the best of our knowledge, this is the first in-depth qualitative account of a telemedicine-supported hybrid primary care model implemented in permanently vacant Hungarian general practices, with direct implications for managing the primary care workforce shortage. A further strength is the standardized nature of the intervention-uniform diagnostic technology and clearly defined nursing roles across all sites-which enhances the transferability of the findings.

The limitations of our study include that it examined the vacant practices of a single, specific disadvantaged region over a relatively short period from June 2025 to January 2026. Although qualitative methodology is excellently suited for uncovering deeper causal relationships, confirming the macroeconomic efficiency and long-term health gains (e.g., improvement in morbidity indicators) of the model requires further, multi-year quantitative and health economics studies. Additionally, this study included only healthcare professionals; patients’ perspectives were not directly assessed. Furthermore, findings are based on perceived changes reported by participants rather than routinely collected outcome indicators. Finally, the hybrid model should be understood as a means of maintaining safe provision where a resident general practitioner cannot presently be recruited, not as a substitute for one. It does not address the underlying causes of the workforce shortage, and its appropriate role is as a bridging arrangement operating alongside, rather than in place of, measures to restore permanent physician staffing. Two further sources of bias warrant consideration. Participation was confined to professionals engaged in delivering the programme, a group that may be predisposed to appraise favourably an innovation in which they are personally invested, and whose accounts may reflect the enthusiasm that commonly accompanies a newly introduced intervention. In addition, data were collected during the first months of operation, so the perceptions reported here are those of an early adaptation period; both the difficulties described, such as unfamiliarity with the devices, and the benefits described, such as the novelty of continuous medical availability, may attenuate or change once the model becomes routine. Finally, no post-hoc member checking was undertaken, so participants did not have the opportunity to comment on the final thematic structure.

## 5. Conclusions

This study examined the implementation of a telemedicine-supported hybrid primary care model in permanently vacant general practices from the perspective of the professionals delivering it. Its findings are accounts of perception and experience rather than measurements of clinical or service outcomes: participants judged the model to be operationally feasible and considered that it improved access, professional support, and the coordination of care, but these are perceived benefits and not demonstrated effects of the intervention.

The principal contribution of the study lies less in establishing that such a model can be operated than in identifying what determines whether it can be sustained. The technological infrastructure proved to be the least problematic component. What constrained the model were the conditions surrounding it: documentation requirements that duplicate effort across paper and digital systems, medical software that does not interoperate, nursing curricula that do not prepare practitioners for delegated telemedicine-supported roles, and a population unaccustomed to triage-based care and therefore inclined to experience it as an obstacle rather than as a means of allocating attention by need. The determinants of successful implementation in a setting of workforce shortage are accordingly organisational, educational, regulatory, and social rather than technological, and interventions that supply equipment without addressing these conditions should not be expected to succeed.

Although conducted in one disadvantaged district of Hungary, the problems addressed here are widely shared: an ageing general practice workforce, unfilled rural posts, and the resulting inequality of access are features of many health systems. The organisational logic of the model, which retains the physical examination at the point of care while delivering clinical judgement remotely and preserves a defined route to in-person assessment, is likely to be more transferable than its specific configuration, which depends on national rules governing nursing scope of practice and on the prior existence of a substitution arrangement. Future research should evaluate patient outcomes, emergency care utilisation, and cost-effectiveness; incorporate the perspectives of patients themselves; and follow the model beyond its initial adaptation period.

## Figures and Tables

**Table 1:** The Heves district in national context: territorial development and primary care provision.

Indicator	Heves District	Hungary (National)
Composite territorial development index (rank among the 197 districts)	24.40 (9th lowest)	46.68 (mean across all districts)
Statutory territorial development classification	Designated for the complex development programme	36 of 197 districts (18.3%) hold this designation
Contracted general practice districts, n	23	6421
Districts without a permanent general practitioner, n (%)	8 (34.8)	956 (14.9)

Rows 1 and 2: Government Decree 290/2014 (XI. 26.) on the classification of beneficiary districts [22], Annexes 2 and 3. Rows 3 and 4: National Health Insurance Fund [13]. National figures refer to all vacant districts (early 2025) and district-level figures to permanently vacant districts (April 2025), so the contrast shown is conservative.

**Table 2:** Deductive codebook.

Category	Code	Definition/Description
1. Access to Care	Difficulty	Lack of primary care, doctor shortages, narrow timeframes of the substitution system, and the resulting “fighting fires.”
	Ease	Improvement of local care, benefits provided by telemedicine points, and patient transport services operated by the Order of Malta.
	Waiting	The length of waiting time spent in the clinic and the determination of patient order.
	Travel	Travel burdens of patients when reaching different care points (e.g., specialist clinic, hospital, on-call service).
2. Application of Telemedicine	Advantages	Positives of remote consultation: continuous availability, increased patient safety, avoidance of unnecessary doctor visits.
	Disadvantages	Clinical uncertainties stemming from the lack of personal contact or patients’ distrust.
	Personal Need	Cases and attitudes where the patient or the doctor insists on a physical, in-person examination.
3. Patient Pathway Organization	Smoothness	Transparency of the care process, retaining patients locally, and avoiding unnecessary institutional visits.
	Institutions	Patient flow between primary care, the Central Clinic, the ambulance service, and specialist care.
4. Professional Support	Professional Support	The sense of security among team members (especially nurses) due to continuous medical background.
	Lack of Professional Support	Professional isolation stemming from the previous/existing system and the excessive self-reliance of assistants.
5. Communication and Relationship	Advantages	Building trust, increased attention, and the emergence of a preventive approach.
	Disadvantages	Authoritarianism, patients’ fear of diagnosis, and low health literacy.
	Patient Info	Passing on instructions, health education of patients, and explaining the new system.
	Within Team	Information flow between GPs, nurses, and the Central Clinic staff.
	Between Care Points	Professional coordination between primary care, on-call services, and the National Ambulance Service.
	Information Gap	Cases where data is lost or the patient has to be asked multiple times about the same issue.
6. Triage System	Advantages	Structured, protocol-based questioning of patients, which improves the accuracy of decision-making.
	Disadvantages	Misunderstandings arising from patient prioritization and changes in the waiting order.

**Table 3:** Inductive codebook.

Category	Code	Definition/Description
1. Infrastructure and Technology	Technical Difficulties	Hardware and software problems: heavy monitors, disconnecting tablets, cabling issues, and fragmented medical software.
2. Processes and Administration	Administrative Burden	Significant time loss and patient impatience resulting from filling out triage sheets and digital documentation.
	Work Organization	Anomalies of the substitution system (e.g., phantom standby) and the excessive, parallel workload of nurses.
3. Perception and Vision of the Pilot	Advantages	Comprehensive positives of the hybrid model (e.g., workload reduction, faster diagnosis).
	Disadvantages	Current limitations of the model, e.g., not yet suitable for primary prevention on its own.
	Development	Participants’ suggestions: unified information technology system, employing more nurses, structural change in primary care.
4. Quality of Care	Satisfaction	Positive experiences of patients and staff, and trust spreading through “word of mouth.”
	Dissatisfaction	The uneven quality of the current substitution system and the vulnerability of local governments.

**Table 4:** Final thematic structure and contributing codes.

Theme	Subtheme	Contributing Codes
1. Access and the organisation of care	Expanded and predictable availability	Access to care: difficulty, ease, waiting, travel; Patient pathway organization: smoothness, institutions
	Coordination between care points	Communication and relationship: between care points, information gap; Perception and vision of the pilot: advantages
2. Professional support and nursing competence	Reduction in professional isolation	Professional support; Lack of professional support; Communication and relationship: within team
	Expanded nursing role and training needs	Application of telemedicine: advantages, disadvantages; Triage system: advantages; Quality of care: satisfaction
3. Administrative and technological constraints	Duplicate documentation and workload	Processes and administration: administrative burden, work organization
	Infrastructure and software fragmentation	Infrastructure and technology: technical difficulties; Perception and vision of the pilot: development, disadvantages
4. Patient adaptation and health literacy	Cultural resistance to structured care	Triage system: disadvantages; Communication and relationship: disadvantages
	Information provision and trust	Communication and relationship: advantages, patient info; Application of telemedicine: personal need; Quality of care: dissatisfaction

**Table 5:** Representative participant quotations illustrating each code of the deductive and inductive codebooks.

Category	Code	Representative Quotation
1. Access to Care	Difficulty	“My experience is that the time window is very narrow. You can absolutely feel that there are too few doctors, …, substitutions in the vacant practices are, by their nature, narrow time slots. It is firefighting, that is how I feel.”
	Ease	“In the practices where the pilot could be implemented, access clearly increased for the patients, …, the practice was effectively open eight hours a day, not only in the previous narrow time slots.”
	Waiting	“Even running a 40-degree fever, if there are 25 people ahead of them, they simply cannot accept that they are 25th in the queue and have to wait for the 24 in front-who also have 40-degree fevers. None of that matters to them, so they just leave.”
	Travel	“They do not have to travel to another town. From the more remote settlements, people would not go all the way to Eger. If I have some problem, I go in to the nurse I know, have a telemedicine consultation, and I am talking to a doctor.”
2. Application of Telemedicine	Advantages	“And with the cameras you can sometimes see better than when we examine in person.”
	Disadvantages	“Making a responsible decision based on a single sense organ, without examinations-forgive me, but that is not a physician’s responsible care, over the telephone.”
	Personal Need	“The young ones or the grandchildren help. Or they come in and arrange it in person.”
3. Patient Pathway Organization	Smoothness	“They do not have to go to another town, …, the Maltese bus comes-cardiology, ultrasound-one street away, …, If I have some problem, I go in to the nurse I know, telemedicine, and I am talking to a doctor.”
	Institutions	“So communication between the professions is still hindered.”
4. Professional Support	Professional Support	“It is a sense of security; they can act much more confidently, …, and they know they can turn to one another, …, I think this system definitely provides that.”
	Lack of Professional Support	“During consultation hours the patient is constantly sitting here and the phone is ringing, …, ‘write a prescription’, ‘I am in hospital’, someone knocks on the door, …, so they are working in parallel. It is inhumane what they do.”
5. Communication and Relationship	Advantages	“There is a chronic patient who lives alone, …, and he is glad that he is really being taken care of; he always gets a referral somewhere, and he does go. And his condition has actually improved since then.”
	Disadvantages	“This disadvantaged population group longs to be healthy just like anyone else, …, but because of their financial situation and health culture, they are terrified to go to the doctor and find out that something is wrong.”
	Patient Info	“After the telemedicine consultation there is still a confirmation between the nurse and the patient: ‘so let us summarize-there will be a referral, you will go here’, …, This happens in every case after the video call is ended.”
	Within Team	“The doctors can talk with the patient so well because the patients are very well prepared. Very strong teamwork is manifested here, and the first half of that teamwork definitely belongs to the nurses.”
	Between Care Points	“Yes, the ambulance service indicated that daytime on-call utilization has decreased.”
	Information Gap	“They [the on-call doctor] cannot see into the system at all-they have no idea what the patient’s underlying conditions are. The patient either knows, or does not, or only partially knows. There is simply no way to retrieve the relevant parameters.”
6. Triage System	Advantages	“The positive of any triage- or guideline-based work is that we do not slip over information, …, if we catch a blood pressure of 190, that improves the quality of care-that is a health gain.”
	Disadvantages	“[Do the patients see the point of triage?] No. What they see in it is that they have to wait longer. ‘Do we still have to write?’”
1. Infrastructure and Technology	Technical Difficulties	“Sometimes the bandwidth is simply not there for the sound to come through in an interpretable quality. The technology is good on one side, good on the other side; it is the connection that is the problem. | “We have to learn [to use it]-we can barely switch it on ourselves. For the younger generation it comes naturally, as they were born into it, but we are 60+.””
2. Processes and Administration	Administrative Burden	“Prescription writing, especially over the telephone: there are 30-35-40, even 50 telephone prescription requests a day, and a triage sheet for each of them, with all of the patient’s regularly taken medicines.”
	Work Organization	“Outside the two-hour consultation, these substitutions include standby time. In this standby time it is in reality the National Ambulance Service that carries out the tasks, …, it is very rare that the substituting GP can get back in time, or at all.”
3. Perception and Vision of the Pilot	Advantages	“The fact that we can treat acute patients in those six hours in which they would previously have called an ambulance or burdened the emergency department, …, especially in terms of scalability, these are important outcomes.”
	Disadvantages	“This programme is certainly not suitable for improving primary prevention on its own at present; that requires a separate team, …, a school-focused, workplace-focused, elderly-focused effort.”
	Development	“A unified medical software is important, …, so that the treating physician can see the patient’s full record and the documentation is done there, not in this fragmented way, being sent back and forth.”
4. Quality of Care	Satisfaction	“I really thank the doctors. They speak with the patients with such patience and expertise, they explain everything, …, our substituting doctors have 56 consultations, they come and go, they run; there is not as much time as our patients get from the video doctors.”
	Dissatisfaction	“There are practices where the paid and expected two to three hours of daily consultation is delivered very reliably, and we also see practices where it is patchy… municipalities are cautious about raising any quality objection against the doctor.”

**Table 6:** Comparison of the HHP with related models of technology-supported primary care in physician-scarce settings.

Model	Setting	In-Person Physician Assessment	Role of the Non-Physician Professional	Technology	Principal Reported Barrier
AGnES programme [24]	Rural northern Germany	Available, the model supports a practising general practitioner	Delegated home visits by practice nurses	Telecare monitoring devices	Regulatory limits on delegation
Whole System Demonstrator [40]	England	Available through the patient’s own practice	Professionals supervise patient self-monitoring	Telehealth and telecare	Professional and patient resistance
Nurse substitution in primary care [38]	Multiple high-income countries	Available within staffed practices	Nurses provide first-contact and ongoing care	Not technology-dependent	Scope-of-practice and training constraints
Nurse-led teletriage [23]	Multiple settings	Not available at the point of triage	Nurse triages by telephone and directs onward	Telephone; decision-support software	No physical assessment at the point of triage
Heves General Practice Pilot (present study)	Permanently vacant rural practices, Hungary	No resident general practitioner in the satellite practices; in-person assessment available at the Central Clinic, to which patients are transported when clinically indicated	On-site triage and device-assisted physical examination	Digital stethoscope, video otoscope, 12-lead ECG, ankle-brachial index meter, teleconsultation platform	Duplicate documentation; software fragmentation; gaps in nursing curricula

## Data Availability

The data presented in this study are available on request from the corresponding author. The data are not publicly available due to privacy and ethical restrictions relating to the interview participants.

## Supplementary Materials

The following supporting information can be downloaded at https://www.mdpi.com/article/10.3390/healthcare14172727/s1, Table S1. Technical specifications of the telemedicine workstation deployed at each participating practice.

## Abbreviations

The following abbreviations are used in this manuscript:

HHP	Heves Haziorvosi Pilot (Heves General Practice Pilot Programme)
APN	Advanced Practice Nurse
ABPM	Ambulatory Blood Pressure Monitor
ECG	Electrocardiogram
GP	General Practitioner
HKR	Hevesi Központi Rendelő (Central Clinic, Heves)
NEAK	Nemzeti Egészségbiztosítási Alapkezelő (National Health Insurance Fund, Hungary)
NNGYK	Nemzeti Népegészségügyi és Gyógyszerészeti Központ (National Centre for Public Health and Pharmacy, Hungary)
NVivo	NVivo Qualitative Data Analysis Software (Lumivero, v15)
