# Supplementary material for: Telemedicine-Supported Hybrid Primary Care in Rural Areas with Healthcare Workforce Shortages: A Qualitative Study from the Heves District, Hungary
Source: Healthcare (Basel). 2026 Aug 26;14(17):2727. doi: 10.3390/healthcare14172727 (PMC13565505; doi:10.3390/healthcare14172727)
Supplement: Supplementary file 1 [file healthcare-14-02727-s001.zip › healthcare-4464097-supplementary.pdf]

Supplementary Material

Telemedicine-Supported Hybrid Primary Care in Rural Areas with Healthcare Workforce Shortages: A Qualitative Study from the Heves District, Hungary

Table S1. Technical specifications of the telemedicine workstation deployed at each participating practice.

**Function**	**Device**	**Manufacturer**	**Reference**
Remote auscultation of heart and lung sounds	Thinklabs One digital stethoscope	Thinklabs Medical LLC, Greenwood Village, CO, USA	[19]
Examination of the ear	Firefly DE500 digital video otoscope	Firefly Global, Belmont, CA, USA	[20]
12-lead electrocardiography, ankle-brachial index and blood pressure measurement	MESI mTABLET integrated diagnostic modules	MESI d.o.o., Ljubljana, Slovenia	[21]
Video teleconsultation	Kameleon teleconsultation camera	Kameleon Kamera Kft., Budapest, Hungary	[22]

An identical workstation was installed at each of the four satellite practices and at the Central Clinic. Remote physician consultations were conducted through a dedicated telemedicine platform, with all devices operated by the on-site nursing staff. Reference numbers correspond to the reference list of the main manuscript.
